# The role of neural precursor cells and self assembling peptides in nerve regeneration

**DOI:** 10.1186/1916-0216-42-60

**Published:** 2013-12-19

**Authors:** Xiao Zhao, Gordon S Liu, Yang Liu, Jian Wang, Kajana Satkunendrarajah, Michael Fehlings

**Affiliations:** 1 Department of otolaryngology – head and neck surgery, University of Toronto, Toronto, Ontario, Canada; 2 Department of Genetics and Development, Toronto Western Research Institute and Spinal Program, Krembil Neuroscience Centre, University Health Network, Toronto, Ontario, Canada; 3 Department of Surgery, University of Toronto, University Health Network, Toronto, Ontario, Canada; 4 Institute of Medical Sciences, University of Toronto, University Health Network, Toronto, Ontario, Canada

## Abstract

**Objective:**

Cranial nerve injury involves loss of central neural cells in the brain stem and surrounding support matrix, leading to severe functional impairment. Therapeutically targeting cellular replacement and enhancing structural support may promote neural regeneration. We examined the combinatorial effect of neural precursor cells (NPC) and self assembling peptide (SAP) administration on nerve regeneration.

**Methods:**

Nerve injury was induced by clip compression of the rodent spinal cord. SAPs were injected immediately into the injured cord and NPCs at 2 weeks post-injury. Behavioral analysis was done weekly and rats were sacrificed at 11 weeks post injury. LFB-H&E staining was done on cord tissue to assess cavitation volume. Motor evoked potentials (MEP) were measured at week 11 to assess nerve conduction and Kaplan meier curves were created to compare survival estimates.

**Results:**

NPCs and SAPs were distributed both caudal and rostral to the injury site. Behavioral analysis showed that SAP + NPC transplantation significantly improved locomotor score p <0.03) and enhanced survival (log rank test, p = 0.008) compared to control. SAP + NPC treatment also improved nerve conduction velocity (p = 0.008) but did not affect cavitation volume (p = 0.73).

**Conclusion:**

Combinatorial NPC and SAP injection into injured nerve tissue may enhance neural repair and regeneration.

## Introduction

Peripheral nerve injury in the head and neck region may result in significant morbidity for patients [1-3]. Advancement in nerve regeneration has been met with varied success, partly related to the various mechanisms of nerve injury such as stretch, laceration, compression, resulting in different degrees of structural disruption and cellular loss [4-7].

Self assembling peptides (SAPs) are bioengineered peptides which assemble into nanofibers *in situ*, forming a scaffold potentially linking damaged nerve segments. SAPs have been shown to enhance axonal regeneration and functional recovery in spinal cord injury models either alone [8], or in combination with a RhoA inhibitor CT04 [9] or a neurite-promoting laminin epitope IKVAV [10]. K2(QL)6 K2 is an SAP composed of gutamine and leucine amino acids which assembles into a β-sheet conformation and is soluble at a pH of 7, very close to physiologic pH. Unlike implanted solid scaffolds, QL6 can be injected into damaged nerve tissue as a liquid, potentially reducing the occurrence of iatrogenic mechanical injury.

Adult neural precursor cells (NPCs) have properties of self renewal and multipotency [11-13]. When harvested from the subventricular zone of the forebrain, it was shown that about 50% of NPCs transplanted into the subacute injured spinal cord tissue differentiated into premature or mature oligodendrocytes [11]. NPC-derived oligodendrocytes enhanced myelination of axons and improved functional recovery. Neurobehavioral recovery of chronically injured spinal cord tissue was also improved with NPC transplantation when chondroitianase ABC was administered via infusion prior to transplantation [14].

We propose that the combinatorial treatment of SAPs and NPCs may enhance recovery after spinal cord injury by providing a scaffold for axonal growth and through enhancing meylination by repopulating oligodendrocytes derived from NPCs. Given the similarities in molecular and anatomic features of the peripheral nervous system (PNS) and central nervous system (CNS), it is tempting to speculate that the therapeutic strategies employed in this study would also be applicable in peripheral nerve regeneration. PNS and CNS employ similar extracellular matrix components involved in development and a large fraction of the PNS cytoplasmic bulk is found within the CNS [15]. Furthermore, both oligodendrocytes and Schwann cells assemble and maintain myelin to promote axonal potential speeds. Our results indicated that the combinatorial treatment of SAP + NPC improved nerve conduction velocity, enhanced functional recovery, and increased the probability of survival after spinal cord injury. However, we did not see a reduction in cavitation or residual volume, potentially due to a survival bias from a high mortality rate in the control group.

## Methods

22 female Wister rats were used in this study (250–300 gm, Charles River Laboratories, Wilmington, MA). SAP + NPC and control groups both had 11 animals. The University Health Network animal care committee approved all experimental protocols in accordance to policies as per the Canadian Council of Animal Care Guide to the Care and Use of Experimental Animals.

### Spinal cord injury

Animals were put under anaesthesia using isoflurane and a 3:1 mixture of oxygen to nitrogen. The surgical site was sterilized using betadine and 70% isopropyl alcohol. T6-T7 was landmarked using the prominent spine of T2 and an incision was made from T4 – T9. Soft tissue was dissected down to the lamina of T6-T7 and laminectomy was performed at T6-T7 exposing the spinal cord. Spinal cord injury was induced using a 35 gm aneurysm clip passed around the cord and snapped closed for 1 minute. This aneurysm clip model of SCI has been well established [16,17].

### SAP-QL6 injection

The primary investigator conducting surgical procedures was blinded to SAP-QL6 and control injection (SAP-QL6: 1% concentration, control: saline). Immediately following spinal cord injury, a Hamilton syringe with a glass capillary needle was used to inject a total of 10 µL of SAP solution or control into the injured nerve tissue at a rate of 0.5 µl/min (5 µl injected 2 mm rostral to injury site and 5 µl injected 2 mm caudal to injury site). The needle was left in nerve tissue for an additional minute after each injection to promote diffusion of SAP-QL6.

### NPC transplantation

NPC isolation and culture was conducted as per Karimi-Abdolrezaee et al. [11]. Rats with T6-7 injury and SAP-QL6 or control injection were pre-treated with immunomodulating agents, minocycline and cyclosporine A, for 24–36 hours pre-operatively. These animals were then put under anaesthesia and the surgical site was reopened, exposing T6-7 cord tissue. NPCs prepared from passage 3–4 neurospheres were mechanically disassociated into single cells and diluted using growth medium (5 × 10^4^/µl) after testing for viability with trypan blue. Control used for NPC transplantation was growth medium. NPCs or control was transplanted into nerve tissue at two points both 2 mm rostrally and caudally to injury site (4 injection sites total) using a Hamilton syringe attached to a glass capillary with total injection volume of 8 µl. To enhance survival of NPCs, an intrathecal catheter was inserted rostral to the injury site and passed caudally past NPC transplantation site. This catheter was attached to an osmotic pump (model 1007D, 0.5 µl/h; Alzat, Cupertino, CA) filled with platelet derived growth factor-AA (1 ug/100 µl, Sigma), basic fibroblast growth factor (3 µg/100 µl, Sigma), and epidermal growth factor (3 µg/100 µl) in a solution containing artificial cerebrospinal fluid, bovine serum albumin (100 µg/ml), and gentamycine (50 µg/ml). The osmotic pump was designed to promote release of these factors over 7 days, thereby enhancing NPC survival. The surgical site was then closed and animals were all treated with minocycline for 1 week and cyclosporine A for the duration of experiments.

### Neurobehavioral testing

To test neurobehavioral recovery after nerve injury, the Basso, Beattie, and Bresnahan (BBB) open field locomotor score was determined by two independent blinded assessors weekly from weeks 1 to 11 post-injury. BBB scoring system is a 21 point scale testing hindlimb recovery through assessment of joint movement, step ability, coordination, and trunk stability. Weekly results were averaged for left and right hindlimbs.

### Electrophysiology

To determine whether SAP + NPC improved nerve conduction characteristics, motor evoked potentials (MEP) were assessed *in vivo*. At week 11 post-injury, a blinded investigator put animals under light sedation using isoflurane and 2 stainless steel subdermal needle electrodes were placed into the biceps femoris. A square pulse (2 mA stimulus of 0.1 ms at 0.13 Hz) was applied to the midline cervical spinal cord and recordings were measured using Keypoint Portable (Dantec Biomed, Denmark). Amplitude was measured between positive and negative peaks and latency was calculated from the initiation of pulse stimulus to first well formed peak. Mean latency and amplitude for each animal was calculated based on average tracing of 200 sweeps.

### H&E processing and measurements

Transcardiac perfusion was performed at 11 weeks post-injury using 4% paraformaldehyde in 0.1 M PBS. Spinal cord tissue with a length of about 2 cm centered at the injury site was extracted and embedded. Serial cross sectioning of the cord was done at 30 micron thickness using a cryostat (Leica CM3050S). Sections were stained with hematoxylin-eosin (HE) and myelin selective pigment luxol fast blue (LFB). Measurements of total cord and cavity volume were assessed by a blinded investigator using Cavalieri Probe (Steroinvestigator software).

### Statistics

Data was assessed using SPSS. Comparison of BBB locomotor scores was done using 2-way ANOVA with post-hoc Bonferroni test. MEP scores and cavitation volume were compared with Student’s t-test. Comparisons of survival between treatment and control groups were done using Kaplan Meier curves.

## Results

### Biodistribution of SAP and NPC in injured spinal cord tissue

**Figure 1:**
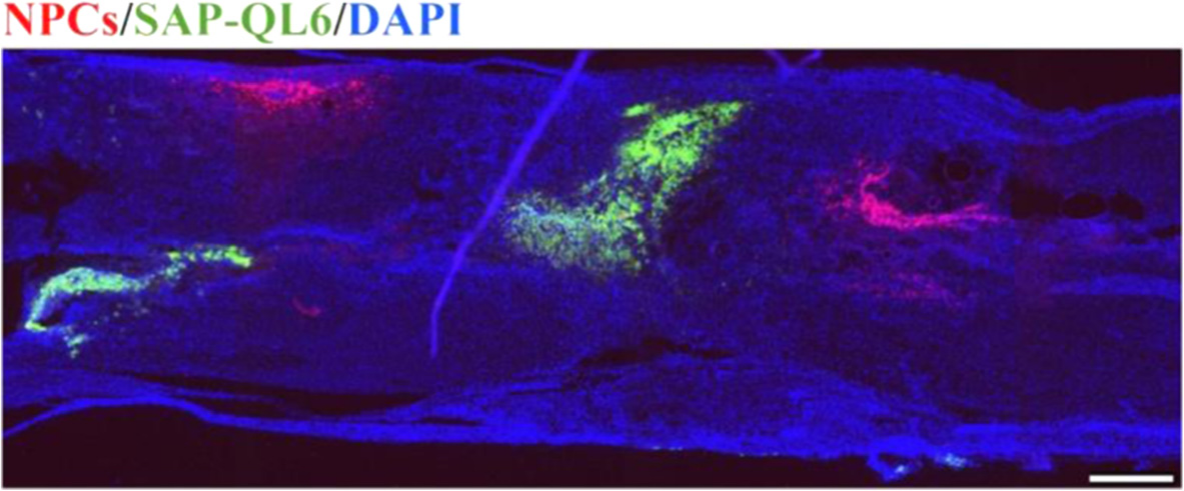
**Biodistribution of SAP and NPC along injured spinal cord.** SAP labelled with FITC was injected immediately after spinal cord injury. NPC labelled with DSred were transplanted 2 weeks after injury. DAPI was used to stain cell nuclei blue. Spinal cord was perfused at 4 weeks post-injury. SAP and NPC were found rostral and caudal to injured cord segment. Scale bar: 600 μm.

To examine the biodistribution of SAPs and NPCs along the injured nerve tissue, we injected SAP-QL6 labelled with FITC immediately post-injury and transplanted NPCs labelled with DSred 2 weeks post-injury. Animals were perfused at day 15 after NPC transplantation. Longitudinal section of the injured spinal cord showed presence of NPCs and SAPs both caudal and rostral to the site of injury (Figure 1).

### SAP + NPC treatment enhances neurobehavioral recovery

**Figure 2:**
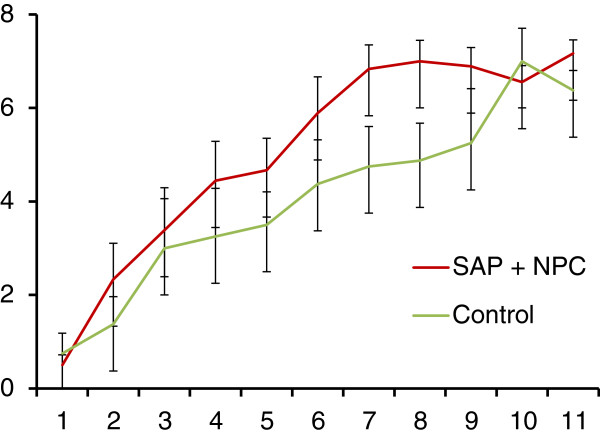
**BBB locomotor score of SAP + NPC treatment and control.** BBB locomotor scores were assessed by 2 independent reviewers from week 1 – 11 post-injury. Both control and SAP + NPC groups exhibited improvements in score over time. SAP + NPC group showed higher mean scores up to week 10. There was a significant difference in scores between the two groups (2-way ANOVA, p = 0.03). Error bars are standard error.

To examine the effect of SAP + NPC treatment on neurologic functional recovery, two independent blinded observers assessed BBB open field locomotor scores from week 1 to 11 post-injury. Both SAP + NPC and control group demonstrated improvements in BBB locomotor scores over time (Figure 2). SAP + NPC treatment resulted in higher mean BBB locomotor scores up to 10 weeks post-injury. At 10 to 11 weeks, there was no difference between the 2 groups in BBB score. Using two way ANOVA, we found a statistically significant difference in BBB locomotor score favouring SAP + NPC treatment group (p < 0.03). Scores for SAP + NPC and control peaked at a mean of 7, suggesting that this may be the limit of functional recovery given the degree of injury.

### SAP + NPC treatment demonstrated improved overall survival

**Figure 3:**
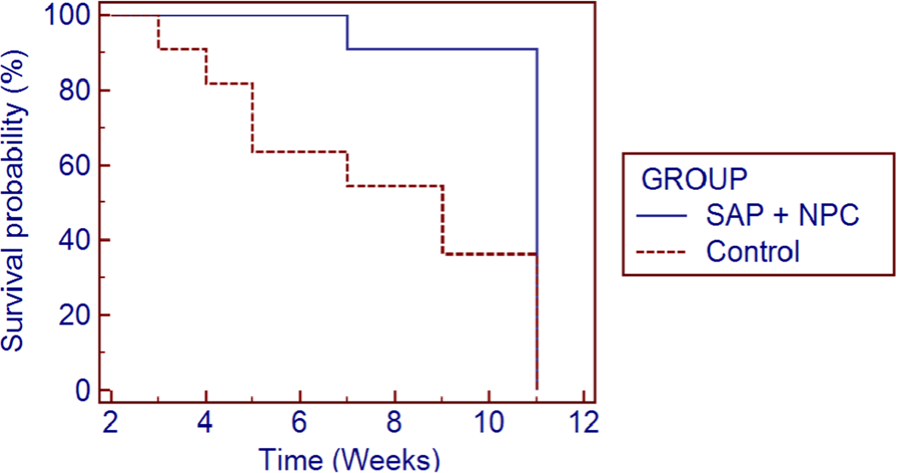
**Kaplan-Meier curve of survival estimates.** Log rank test was significant between the two groups (p < 0.008). Percentage of animals surviving at week 11 was 36% (4/11) for control group and 91% (10/11) for SAP + NPC treatment group.

Poor motor and automatic function following spinal cord injury results in an increased risk of mortality [18]. It was observed that a large number of control animals were dying secondary to UTIs relative to SAP + NPC group. To determine whether SAP + NPC treatment resulted in an increase in overall survival, we plotted a Kaplan-Meier curve comparing survival between SAP + NPC and control groups (Figure 3). There was a statistically significant difference in log rank test between the two groups with 36% of control (4/11) and 91% of SAP + NPC (10/11) treated animals surviving to week 11 (p = 0.008). Two animals died independent of a UTI. One animal in the SAP + NPC group had to be sacrificed due to neuropathic pain, and 1 animal in the control group died due to an intra-abdominal abscess, possibly secondary to intraperitoneal minocycline injection.

### SAP + NPC treatment did not result in less cavitation

**Figure 4:**
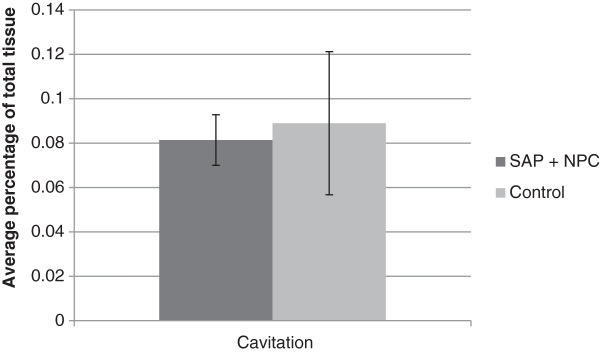
**Cavitation volume percentage.** Cord tissue was collected at week 11 post-injury and cross sections stained with LFB-HE. Percentage cavitation was calculated based on cavity volume divided by total cord volume for each animal. There was no statistical difference between SAP + NPC and treatment groups in percentage cavitation (p = 0.73).

To determine whether SAP + NPC treatment was correlated with a reduction in cavitation, injured cord tissue was stained with LFB-HE at 11 weeks post-injury. A blinded assessor then assessed whole cord and cavity volume. There was no statistically significant difference between SAP + NPC treatment and control cavity volume (8.14% vs 10.66%) by Student’s t-test (p = 0.73) (Figure 4).

### SAP + NPC treatment improved motor evoked potentials

NPCs have been shown to differentiate into oligodendrocytes and increase myelination of axons. To determine whether nerve conduction was enhanced by SAP + NPC treatment, MEPs were assessed at 11 weeks post-jury. SAP + NPC treatment resulted in decreased latency compared to control (20.68 +/- 5.27 vs 30.53 +/- 7.54), indicating a faster velocity of conduction (p = 0.008).

## Discussion

Damage to cranial nerves, whether through a disease process or by iatrogenic injury, is uncommon but is associated with significant morbidity [1-3]. In the head and neck, injury to the facial nerve as well as CN9-12 can result in an array of debilitating conditions ranging from facial deformity to swallowing difficulties, dysphonia, to airway compromise. Multiple injury-associated defects, such as loss of structural and cellular components, may impede the success of single modality treatments aimed at nerve regeneration. As such, multimodality strategies targeted at repopulating myelinating cells and reconstructing structural support through bioengineered scaffolds may enhance nerve regeneration. In this study, we found that the combinatorial treatment of SAP-QL6 and NPCs enhanced neurobehavioral recovery, increased conduction velocity, and improved survival.

The injury model used in this study employed a modified aneurysm clip applied to the spinal cord at T6-7 resulting in a contusion, compression, and stretch injury. This mechanism may be similar to iatrogenic nerve injury as compression and stretch are two of the main mechanisms of peripheral nerve injury [7]. Although the spinal cord was used as a nerve injury model, the similarities between the PNS and CNS suggest that the structural support afforded by SAP-QL6 injection and potential for myelination by NPC transplantation could benefit PNS regeneration, such as cranial nerve palsies in otolaryngology. It has been shown that NPCs, when embedded in a collagen gel and wrapped around an injured segment of sciatic peripheral nerve, differentiate into Schwann-like supporting cells and increased the number and diameter of myelinated fibers [19]. On the other hand, transplantation of Schwann cells into the injured spinal cord was found to enhance meylination and improve hindlimb function [20].

SAP + NPC treatment improved BBB locomotor score and decreased conduction latency, which, taken together, demonstrate a benefit of the combinatorial treatment in neurological functional recovery. Interestingly, BBB locomotor scores of the control group matched SAP + NPC treatment at 10 and 11 weeks post-injury. It could be that SAP + NPC treatment improved the rate of recovery, but did not affect overall functional recovery. However, SAP + NPC treated animals exhibited lower MEP latency at week 11 post-injury, suggesting that BBB locomotor score may not account for subtle changes in function. SAP + NPC treated animals scored in the range of 6 to 8 and an increase in score from 8 to 9 is the difference between coordinated full hindlimb movement and weight bearing, which would require significantly more complex recovery. Finally the lack of difference in BBB scores at week 10 and 11 may be due to a survivor bias, as there were significantly more deaths in the control group (Figure 3).

The high mortality rate in the control group was likely secondary to an increased risk of urinary tract infections (UTIs). Spinal cord injury at level T6-7 results in a neurogenic bladder and all but two deaths in this study were attributed to UTIs. The risk of sepsis and death secondary to UTI was likely increased as all animals were given immunosuppressive drugs for the entire duration of the experiments. UTI was diagnosed based on a combination of cloudy discharge, foul smelling urine, and lethargy. Any suspected UTI was treated empirically with amoxicillin. Given that the internal and external sphincter of the bladder are under autonomic and voluntary control respectively, the significantly lower risk of mortality with SAP + NPC treatment suggests autonomic and/or somatic recovery of bladder function was improved in the treatment group compared to control.

In this study, we did not find a significant difference in cavity volume between SAP + NPC treatment and control groups. It could be that there was a survival bias in favour of the control group, as animals with more cavitation were likely to have a higher risk of mortality. Even so, the improvements in BBB locomotor score and MEP latency suggest that a reduction in cavity volume was not necessary for functional recovery.

## Conclusion

SAP + NPC treatment was found to improve BBB locomotor scores, nerve conduction velocity, and survival. Taken together, SAP + NPC may improve functional recovery after nerve injury. Given the similarities between the PNS and CNS, this combinatorial treatment strategy may hold promise for regeneration of the PNS. Future work will build on this pilot study comparing SAP + NPC treatment to SAP and NPC treatment alone, as well as using this treatment strategy for a peripheral nerve injury model, such as the facial or sciatic nerve.

## Competing interests

SAP-QL6 was supplied by Covidien. The authors have no competing interests.

## Authors’ contributions

XZ preformed the nerve injury, self assembling peptide injection, neuroprogenitor cell transplantation, statistical analysis, and drafted the paper. GSL prepared tissue sections and analyzed hematoxylin-esoin slides. YL provided technical guidance for surgical techniques and prepared self-assembling peptide for use. JW cultured neuroprogenitor stem cells and prepared cells for transplantation. KS performed electrophysiology measurements. MGF supervised the project and provided technical and conceptual guidance. All authors read and approved the final manuscript.
